# Bacterial effector kinases and strategies to identify their target host substrates

**DOI:** 10.3389/fmicb.2023.1113021

**Published:** 2023-02-10

**Authors:** Brendyn M. St. Louis, Sydney M. Quagliato, Pei-Chung Lee

**Affiliations:** Department of Biological Sciences, College of Liberal Arts and Sciences, Wayne State University, Detroit, MI, United States

**Keywords:** post-translational modifications, phosphorylation, secretion systems, signal transduction, host-pathogen interactions, phospho-proteome, bacterial pathogenesis

## Abstract

Post-translational modifications (PTMs) are critical in regulating protein function by altering chemical characteristics of proteins. Phosphorylation is an integral PTM, catalyzed by kinases and reversibly removed by phosphatases, that modulates many cellular processes in response to stimuli in all living organisms. Consequently, bacterial pathogens have evolved to secrete effectors capable of manipulating host phosphorylation pathways as a common infection strategy. Given the importance of protein phosphorylation in infection, recent advances in sequence and structural homology search have significantly expanded the discovery of a multitude of bacterial effectors with kinase activity in pathogenic bacteria. Although challenges exist due to complexity of phosphorylation networks in host cells and transient interactions between kinases and substrates, approaches are continuously being developed and applied to identify bacterial effector kinases and their host substrates. In this review, we illustrate the importance of exploiting phosphorylation in host cells by bacterial pathogens *via* the action of effector kinases and how these effector kinases contribute to virulence through the manipulation of diverse host signaling pathways. We also highlight recent developments in the identification of bacterial effector kinases and a variety of techniques to characterize kinase-substrate interactions in host cells. Identification of host substrates provides new insights for regulation of host signaling during microbial infection and may serve as foundation for developing interventions to treat infection by blocking the activity of secreted effector kinases.

## Introduction

The infection process of bacterial pathogens is a fascinating display of how microbes utilize their arsenal to subvert host defenses. On the defensive side, host cells need to detect and promptly signal the presence of invading pathogens in order to generate proper responses against infection. To evade host responses, gram negative bacterial pathogens use specialized secretion machinery such as type III or type IV secretion systems (T3SS/T4SS), to translocate effector proteins across the bacterial envelope and plasma membrane of targeted hosts (Costa et al., 2015; Bienvenu et al., 2021; Horna and Ruiz, 2021; Grishin et al., 2022; Sanchez-Garrido et al., 2022). After being deployed into the host cytoplasm, these bacterial effectors can target different host proteins and consequently alter signaling pathways to modulate host responses, which is critical for establishing successful infection by the pathogen (Alto and Orth, 2012; Macho and Zipfel, 2015; Ratner et al., 2017; Pinaud et al., 2018).

Post-translational modifications (PTMs) play key roles in regulating protein functions and signal transduction. Protein phosphorylation is one of the most commonly used PTMs for signal transduction in organisms ranging from bacteria to humans (Ubersax and Ferrell, 2007). Protein phosphorylation involves transferring the γ-phosphate of adenosine triphosphate (ATP) onto tyrosine, serine, or threonine residues of a substrate protein. This process is catalyzed by kinases and the phosphate group covalently conjugated on the substrate can be reversibly removed by phosphatases. Phosphorylation is critical in a multitude of signaling pathways that are crucial for the cells, for example, cell cycle initiation through phosphorylation of PP-1, insulin signaling through phosphorylation of AKT, and organ development through phosphorylation of YAP/TAZ (Kwon et al., 1997; Van Weeren et al., 1998; Chen et al., 2020). It is not surprising that host innate immunity is also highly regulated by protein phosphorylation and kinase targeting is a promising field for therapeutics (Gaestel et al., 2009). Thus, many bacterial pathogens have evolved to utilize protein phosphorylation to hijack host signaling pathways and gain control of their host cellular processes for replication and survival (Grishin et al., 2015; Tegtmeyer et al., 2017; Park et al., 2019). Despite the versatility of kinases in signaling networks and diversity of their substrates, many eukaryotic kinases share amino acid sequence homology in their kinase domains, particularly with highly conserved functional motifs participating in phosphate transfer. Structurally, kinases also share high degrees of similarity. Eukaryotic kinase domains, typically consisting of 12 subdomains, display structural similarity by forming a catalytic cleft surrounded by an N-terminal lobe and C-terminal lobe (Hanks and Hunter, 1995; Canova and Molle, 2014). These similarities allow pathogens to mimic a wide range of host kinases and phosphorylate target substrates during infection (Grishin et al., 2015).

Genome and amino acid sequence comparisons have discovered that many bacterial pathogens, including *Salmonella*, *Shigella*, *Yersinia*, and *Legionella*, encode effectors with primary sequence homology to eukaryotic kinase domains (Table 1; orange shade). In addition, with the increasing number of solved kinase structures and the development of structural prediction tools, several bacterial effectors with high structural similarity but limited primary sequence homology to eukaryotic kinases have been recently identified as a new class of effector kinases, including LegK7, XopC2, HopBF1, and VopG (Table 1; blue shade). Advancements in identifying effector proteins of bacterial secretion systems *via in silico* analyses have also allowed more effector kinases to be discovered. For example, a bioinformatic search of effector repertoires in sequenced genomes from *Legionella* species discovered protein kinase activity as the second largest functional domain predicted in the effectors and hundreds of putative effector kinases were identified (Gomez-Valero et al., 2019).

**Table 1:** Bacterial effector kinases, currently identified targeted host pathway, and substrate identification methods.

Effector *Organism*	Interacting Protein	Targeted Host Pathways	Host Substrates	Methods	References
YpkA (YopO) *Y. pseudotuberculosis*	Actin	Cytoskeleton Rearrangement	Gɑq, Otubain-1, gelsolin (+others)	Affected pathway, chemical genetics, SILAC	Juris et al. (2000, 2006); Navarro et al. (2007)
SteC *S. enterica*		Cytoskeleton Rearrangement	MEK	Affected pathway	Poh et al. (2008)
LegK1 *L. pneumophila*		NFκB	IκB	Affected pathway	Ge et al. (2009)
LegK2 *L. pneumophila*		Cytoskeleton Rearrangement	ARPC1B, ARP3	Protein–protein interaction	Michard et al. (2015)
LegK4 *L. pneumophila*		Host translation	HSP70	Chemical genetics	Moss et al. (2019)
PknG *M. tuberculosis*	RAB14	Lysosome trafficking; Autophagy	TBC1D4	Affected pathway	Ge et al. (2022)
OspG *S. flexneri*	UbcH5b, Ubiquitin	NFκB	?	?	Kim et al. (2005), Pruneda et al. (2014)
NleH1/2 *E. coli*		NFκB, Micropillus formation	CRKL, EPS8L2	Proteomic microarray screen; Phosphoproteome analysis	Pham et al. (2013), Pollock et al. (2022)
Lem28 *L. pneumophila*	IP6	LCV?	?	?	Anderson et al. (2015), Sreelatha et al. (2020)
LegK7 *L. pneumophila*	MOB1	Hippo pathway	MOB1	Proteomic microarray screen	Lee et al. (2017)
XopC2 *X. oryzae*		Stomatal Closure	OSK1	Affected pathway	Wang S. et al. (2021)
HopBF1 *P. syringae*		Immune sensing	HSP90	Protein–protein interaction	Lopez et al. (2019)
VopG *V. parahaemolyticus*	?	NFκB?	?	?	Plaza et al. (2021)

?, currently unidentified.

One of the key steps in understanding the role of effector kinases in microbial pathogenesis is identification of their host substrates. This identification allows for the characterization of effector kinase function at a molecular level and provides a foundation for developing interventions to treat infection by interfering with the actions of effector kinases. However, it is particularly challenging due to the transient nature of kinase-substrate interactions and the complexity of phosphorylation networks since endogenous host kinases also catalyze phosphorylation. In this article, we document approaches including genetic, biochemical, proteomic, and high-throughput screening techniques that have been developed and applied to identify host substrates of bacterial effector kinases. Like eukaryotic kinases, activity and substrate specificity of effector kinases can be regulated by their interacting partners, adding another layer of research interest in studying molecular mechanisms by which effector kinases target host proteins. Thus, we also discuss how effector kinases are regulated by their trans-kingdom interacting partners in host cells.

## Substrate identification based on affected host pathways

**Figure 1:**
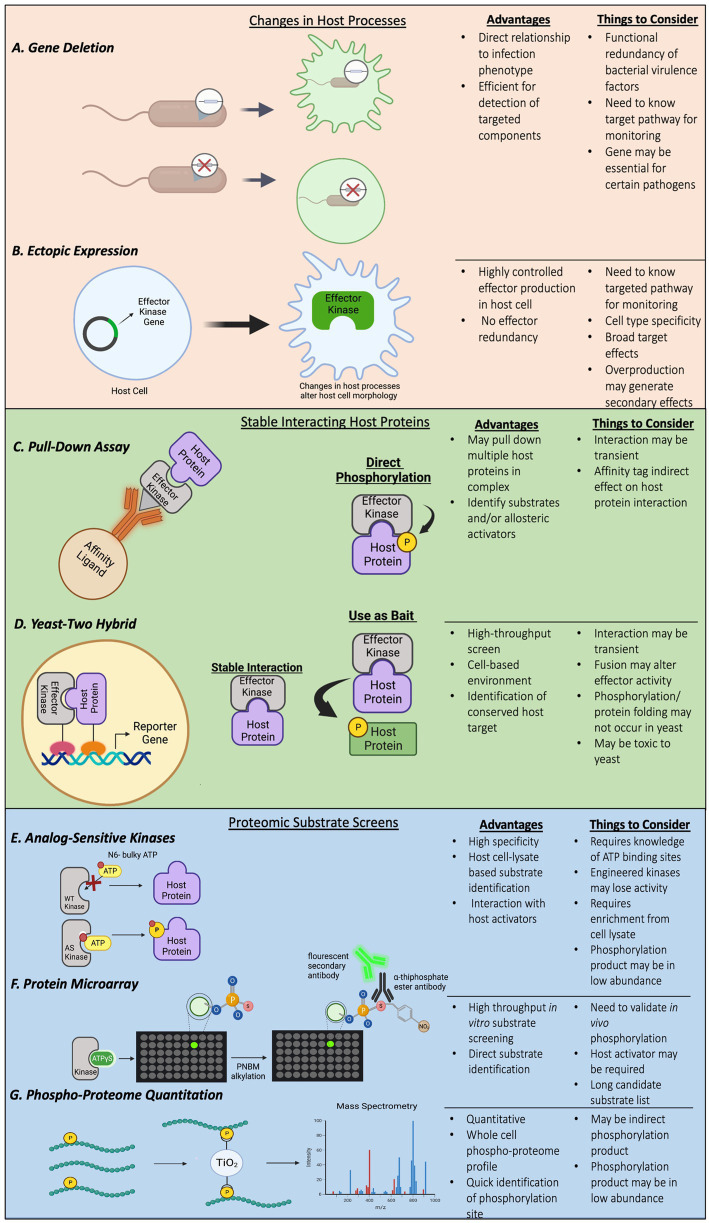
**(A–G)** Examples of methods and techniques used to identify bacterial effector kinases and their host substrates.

Bacterial pathogens use effectors to subvert host defense, including phagocytosis and inflammatory gene expression (Alto and Orth, 2012). Characterizing functions of effector proteins in bacterial pathogenesis can be achieved by comparing responses in host cells infected by a wild-type bacterium or a mutant with the effector gene of interest deleted (Figure 1A). Alterations in targeted cellular processes, such as cytoskeleton rearrangement, maturation of pathogen-containing vacuoles, and activation of inflammatory signaling are commonly monitored. As an alternative approach to simplify the complexity of infection process and potential redundancy of virulence factors produced by the pathogens, ectopically expressing bacterial effectors in host cells may generate similar effects as observed in infected host cells (Figure 1B). Both approaches have been used to characterize bacterial effector kinases and identify specific host protein substrates. Once the host responses or pathways affected are identified, it would suggest the components in the affected pathway are likely targeted by the effector, which allows researchers to narrow down the search for candidate host substrates of effector kinases.

### *Yersinia* YpkA phosphorylates host Gɑq to impair phagocytosis

*Yersinia* spp., including *Y. pestis, Y. pseudotuberculosis and Y. enterocolitica*, use a T3SS to translocate effector proteins into host cells (Cornelis, 2002). YpkA, also called YopO in *Y. pseudotuberculosis*, is a T3SS effector that is required for the virulence of *Yersinia spp.* (Galyov et al., 1993). The N-terminus of YpkA exhibits primary sequence homology and structural similarity to eukaryotic serine/threonine kinases, such as the family of RhoA-binding kinases, and the C-terminus contains a Rho-GTPase binding domain that interacts with host small GTPases RhoA and Rac, important for cytoskeletal organization (Dukuzumuremyi et al., 2000). The kinase activity of YpkA is critical for immediate *Yersinia pseudotuberculosis* survival in a cell-based infection model and virulence in a mouse model (Wiley et al., 2009). YpkA induces morphological changes in infected host cells and production of YpkA in mammalian cells disrupts arrangement of actin filaments, a phenomenon that requires both the kinase activity and C-terminus of YpkA (Juris et al., 2000). This destabilization likely contributes to an impairment of phagocytic ability of the host and is dependent on the interaction between YpkA and host monomeric Rho-GTPases (Grosdent et al., 2002). These observations suggest that YpkA targets host proteins involved in actin cytoskeleton regulation.

**Figure 2:**
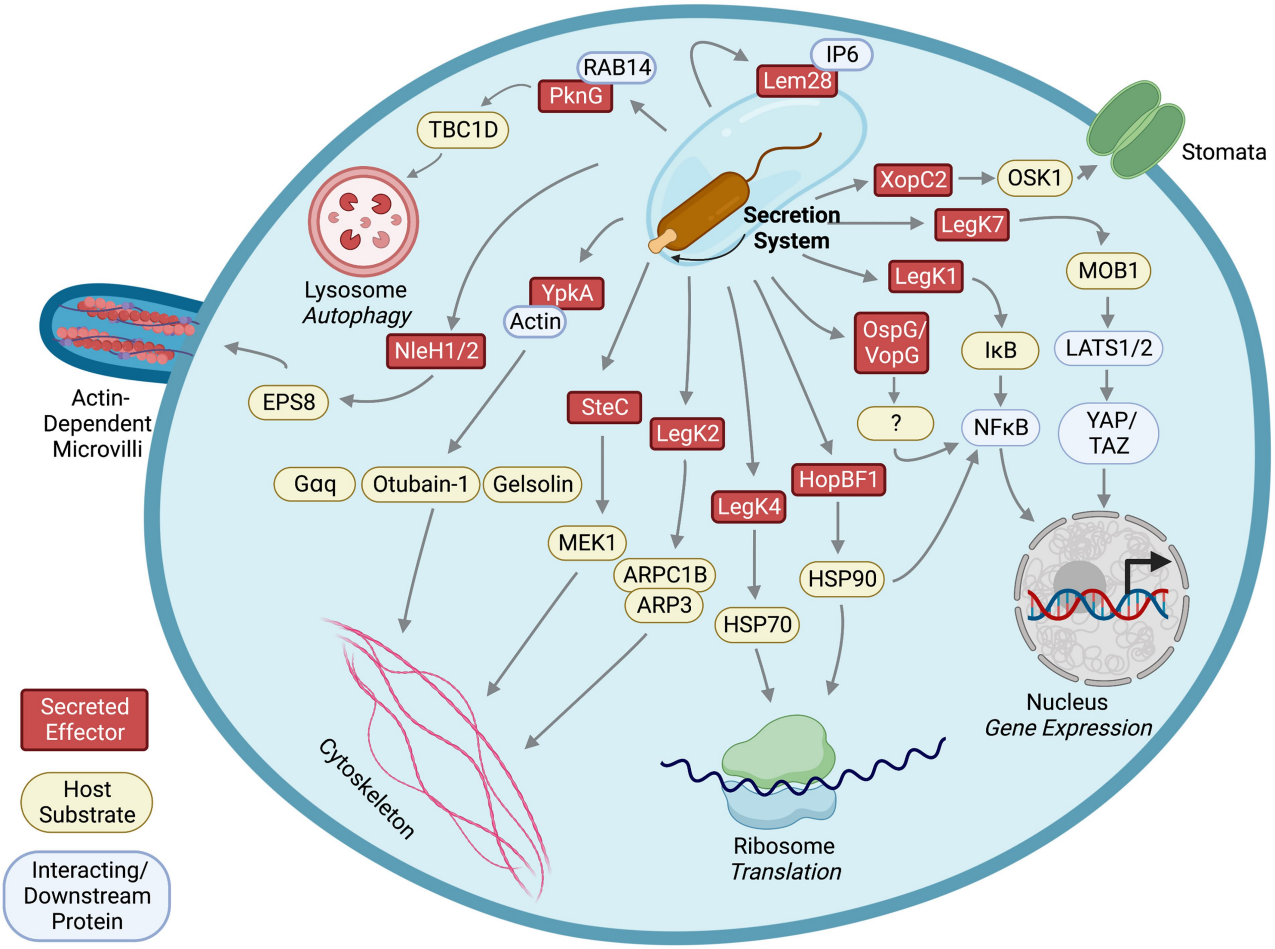
Identified effector kinases, their host substrates, interacting partners, and downstream affected pathways.

Interestingly, although YpkA binds to both GTP-and GDP-bound RhoA, a constitutively active RhoA mutant which mimics the GTP-bound status rescues the alternation of actin stress fibers by YpkA (Navarro et al., 2007), suggesting that the host target of YpkA acts upstream of RhoA and that YpkA manipulates this host target to generate suppressive effects on RhoA. Because G heterotrimeric proteins, Gα12/13 and Gαq, are regulators for RhoA activity in G-protein-coupled receptor (GPCR)-mediated actin rearrangement, screening GPCR agonists in human cells ectopically expressing YpkA revealed that YpkA inhibits activity of Gɑq to suppress RhoA-mediated stress fibers formation induced by the GPCR agonists (Navarro et al., 2007). This inhibition effect is due to YpkA’s ability to phosphorylate and interact with Gɑq. Specifically, YpkA phosphorylates the serine-47 residue on Gɑq to prevent the Gɑq-GTP interaction, thereby inhibiting Gɑq activation and altering actin filament structure in the host cells (Figure 2). Interfering with actin rearrangement by YpkA leads to a disruption of phagocytosis of *Y. psuedotuberculosis* by host cells (Navarro et al., 2007). In addition to affected host pathway analysis identifying Gɑq as a YpkA substrate involved in cytoskeleton rearrangement, other approaches, such as stable isotope labeling of amino acids in cell culture (SILAC; Lee et al., 2015) and chemical engineering of YpkA (Juris et al., 2006) also identified additional cytoskeleton regulators as substrates of this effector kinase, which we will discuss in later sections.

### *Salmonella* SteC phosphorylates host MAP kinase MEK1 to promote SCV formation

When invading host cells, *Salmonella enterica* Typhimurium forms a *Salmonella-*containing vacuole (SCV) for replication. Two T3SS, named *Salmonella* pathogenicity island I and II (SPI-1, −2), are essential for *S. enterica* infection (Chakravortty et al., 2005; Jennings et al., 2017; Galán, 2021). Formation of the SCV requires effectors secreted by the *Salmonella* T3SS and is associated with an F-actin meshwork surrounding the SCV. *Salmonella* SteC is a T3SS SPI-2 effector that shares sequence homology with human kinase Raf-1 (Poh et al., 2008). Formation of F-actin meshwork during *Salmonella* infection depends on SteC and its kinase activity. Consistently, ectopic expression of SteC in mammalian cells induces F-actin rearrangement similar to the effects caused by overproduction of host Rho-associated protein kinase (ROCK), suggesting that SteC likely affects host pathways regulated by ROCK to alter actin filament structure and conformation (Poh et al., 2008).

In mammalian cells, ROCK and myosin light chain kinase (MLCK) phosphorylate myosin light chain (MLC) to activate Myosin II, a key regulator of actin organization and cross-linking (Vicente-Manzanares et al., 2009). It was shown that phosphorylated Myosin IIB is recruited to the SCV and F-actin meshwork in an SteC-dependent manner during *Salmonella* infection (Odendall et al., 2012). Furthermore, siRNA-mediated knockdown experiments showed that MLCK is required for SteC-induced F-actin rearrangement, indicating that SteC activates MLCK to phosphorylate Myosin IIB. Since extracellular signal-regulated kinases (ERKs) regulate MLCK, and SteC shares sequence similarity to host Raf which participates in the Raf/MEK (MAPK/ERK kinases)/ERK pathway, further investigation into this signaling pathway revealed that SteC directly phosphorylates MEK1 *in vitro* at serine-200. Phosphorylation of this residue contributes to MEK1 activation by promoting autophosphorylation (Odendall et al., 2012). Thus, *Salmonella* SteC phosphorylation of host MEK1 activates the MEK/ERK/MLCK pathway to modulate Myosin IIB-mediated F-actin rearrangement (Figure 2). Interestingly, an *S. enterica steC* mutant replicates more efficiently than a wildtype strain (Odendall et al., 2012), suggesting that SteC may modulate intracellular replication of the bacterium.

### *Legionella* LegK1 phosphorylates Host IκB to activate NFκB signaling

The NFκB pathway plays a central role in controlling inflammatory gene expression and host cell survival during microbial infection (Rahman and McFadden, 2011). *Legionella pneumophila* induces nuclear accumulation of the NFκB transcription factors and expression of NFκB-regulated genes in a T4SS-dependent manner (Losick and Isberg, 2006; Abu-Zant et al., 2007). Blocking NFκB nuclear translocation causes host cell death and prevents replication of *L. pneumophila* within host cells (Losick and Isberg, 2006). These results suggest that *Legionella* T4SS effector (s) manipulate host NFκB expression and translocation to promote intracellular replication of the bacterium. Because *L. pneumophila* encodes ~300 T4SS effectors that share functional redundancy (Ensminger and Isberg, 2009; Ensminger, 2016), it is challenging to use genetic knockout approaches to identify *L. pneumophila* effectors that affect specific host cell pathways. Thus, NFκB-luciferase reporter assays combined with ectopic expression of *Legionella* effector libraries in human embryonic kidney cells (HEK cells) were used (Ge et al., 2009; Losick et al., 2010). Two *L. pneumophila* effectors, LnaB and LegK1, highly activate NFκB-controlled luciferase expression and nuclear translocation of NFκB upon production in the human cells (Ge et al., 2009; Losick et al., 2010), suggesting that these effectors target the NFκB pathway.

LegK1 is one of the four *L. pneumophila* effectors that have primary sequence homology to eukaryotic serine/threonine kinases (Hervet et al., 2011). Expression of a LegK1 catalytically inactive mutant fails to stimulate NFκB-driven luciferase expression and nuclear translocation in HEK cells (Losick et al., 2010). Therefore, LegK1 likely modulates the NFκB pathway *via* phosphorylation of host proteins. The NFκB transcription factor consists of two protein subunits, p50 and RelA, and is sequestered in the cytoplasm by binding to inhibitors of κB (IκB). Phosphorylation of IκB by IκB kinases (IKKs) results in the ubiquitination and proteasomal degradation of IκB, allowing translocation of NFκB to the nucleus (Rahman and McFadden, 2011). Subsequently, expression of LegK1 in HEK cells also increases IκBɑ phosphorylation and the addition of purified LegK1 into host cell lysate results in IκBɑ phosphorylation and processing independent of cellular IKKs (Ge et al., 2009). Finally, *in vitro* kinase assays revealed that LegK1 directly phosphorylates IκBɑ and other members of the IκB family, such as IκBβ, IκBε, and p100 (Ge et al., 2009), indicating that *L. pneumophila* LegK1 may phosphorylate a multitude of IκB proteins to regulate the host NFκB pathway (Figure 2). Mutants with individual *legK1* or *lnaB* genes deleted replicated within host cells as efficiently as wildtype *L. pneumophila* did, a common phenomenon due to functional redundancy of the large effector repertoire (Ge et al., 2009; Losick et al., 2010). Interestingly, a *legK1/lnaB* double mutant did not exhibit defects in pathogenesis within host cells (Losick et al., 2010), suggesting that other *L. pneumophila* effectors might compensate for the loss of LegK1 and LnaB.

### *Xanthomonas* XopC2 phosphorylates host OSK1 to prevent stomatal closure in plants

Similar to bacteria that use effector kinases to infect their animal hosts, bacterial pathogens also interfere with plant hosts by using secretion systems to deliver effectors (Feng and Zhou, 2012), including kinase effectors. XopC2 of *Xanthomonas oryzae pv. oryzicola* has been characterized as a T3SS effector kinase that is capable of countering plant defense (Wang S. et al., 2021). Traditional primary sequence or structural homology search tools, such as Pfam and Phyre, did not identify kinase motifs or kinase folds in XopC2. However, HHPred, used for detecting remote protein homology, predicted a putative kinase motif within XopC2. Subsequent *in vitro* kinase assays demonstrated that XopC2 possesses kinase activity (Wang S. et al., 2021), suggesting that XopC2 is an atypical kinase. Importantly, transgenic rice plants expressing XopC2 are more susceptible to *X. oryzae pv. oryzicola* with more severe disease lesions, indicating a role of this effector in suppressing host defense. Moreover, kinase activity of XopC2 is required for this suppression effect (Wang S. et al., 2021). Therefore, XopC2 phosphorylates a host protein to counteract anti-bacterial activities in infected plants.

In plants, stomata are pores for gas exchange and are major targets for pathogens as entrances to invade plant tissues. Plants close the stomal pores as a defense against invading pathogens. Defects in stomal closures were observed in rice plants expressing XopC2 upon infection, indicating regulation of stomal closures could be targeted by XopC2 (Wang S. et al., 2021). Stomal defense is negatively controlled by jasmonic acid (JA) signaling, and activation of JA signaling is mediated by degradation of a family of inhibitory proteins, JAZs. Investigation of JAZ degradation revealed enhanced JAZ9 ubiquitination by XopC2 (Wang S. et al., 2021). The SCF (SKP, cullin, and F-box E3 ubiquitin-protein ligase complex) is majorly involved in the regulation of stomatal closure *via* a ubiquitination-mediated proteasomal degradation pathway (Howe et al., 2018). Under normal conditions, OSK1, the SCF adaptor protein, is loosely associated with COI1b, promoting low levels of JAZ ubiquitination, degradation, and JA signaling. Basal or low JA signaling allows for maintenance of stomatal closure and consequent prevention of bacterial entry and infection. Further investigations into the effect of XopC2 on SCF ubiquitination complex unveiled that XopC2 directly phosphorylates OSK1 (Wang S. et al., 2021; Figure 2). XopC2-dependent OSK1 phosphorylation at serine-53 enhances its binding to the jasmonate receptor COI1b, thereby promoting the ubiquitination and proteasomal degradation of JAZ (Wang S. et al., 2021). Complete degradation of JAZ activates JA-signaling, suppressing stomatal closure, allowing bacterial entry and promoting *Xanthomonas* infection and disease susceptibility in rice plants. Like YpkA interacting with its host substrate Gɑq, pulldown assays showed that XopC2 also interacts with OSK1 (Wang S. et al., 2021), demonstrating that bacterial effector kinases utilize stable interactions to phosphorylate host substrates.

### *Mycobacterium* PknG phosphorylates host TBC1D4 to prevent autophagosome maturation

*Mycobacterium tuberculosis* is an intracellular pathogen that is generally confined to macrophage phagosomes upon infection. As an anti-microbial defense, the phagosomes undergo a maturation process and fuse with lysosomes to eliminate the pathogen. As observed with the pathogen-host molecular arms race, *M. tuberculosis* is adept at escaping these maturing phagosomes to enter the cytosol, simultaneously allowing their recapture by host autophagosomes in the cytosol (Russell et al., 1997; Simeone et al., 2012; Hu et al., 2020). Like other bacterial pathogens, *M. tuberculosis* have evolved to secrete effector proteins, including an effector kinase, PknG, to counteract host defense and promote infection (Walburger et al., 2004; Nicholson and Champion, 2022). The kinase activity of PknG is essential to *M. tuberculosis* pathogenicity (Walburger et al., 2004) and has therefore been a potential antibiotic target for tuberculosis treatments (Gil et al., 2013; Swain et al., 2021). Studies in PknG’s role in pathogenesis show that this effector contains versatile activities, such as E3-ubiquitin ligase activity for host protein ubiquitination (Wang J. et al., 2021; Shariq et al., 2022) and regulatory activity for metabolism, virulence, and stress response within *M. tuberculosis* (Khan et al., 2017; Rieck et al., 2017; Lima et al., 2021). In this section, we focus on a newly identified host protein that is phosphorylated by PknG and approaches used to identify this host substrate.

The kinase activity of PknG is required to suppress fusion of lysosomes to phagosomes and autophagosomes, where a catalytically inactive mutant loses the ability (Ge et al., 2022). Interestingly, PknG promotes formation of autophagosome as suggested by increased LC3 puncta, a key scaffold protein and marker for autophagosomes, in host cells infected with *M. tuberculosis* (Ge et al., 2022). Similar to preventing lysosomal fusion, PknG kinase activity is required for promoting autophagosome formation, suggesting that a host protein involved in autophagosome formation is targeted by PknG. A yeast two-hybrid screen in mouse cDNA library identified host AKT interacts with PknG (Ge et al., 2022). Although PknG inhibits AKT activation by this interaction to promote autophagosome formation, phosphorylation of AKT by PknG is not shown. Instead, another host protein interacting with PknG, RAB14, emerged from the yeast two-hybrid screen, serving as a lead to identification of host substrates for PknG (Ge et al., 2022). RAB14, a small GTPase that is known to regulate vesicle trafficking and formation of *M. tuberculosis*-containing phagosomes (Kyei et al., 2006), is initially targeted and bound by PknG to maintain its GTP-bound state in order to prevent lysosomal fusion. This process is dependent on kinase activity of PknG, but like AKT, RAB14 is not phosphorylated by PknG (Ge et al., 2022). To keep RAB14 in GTP-bound state, PknG phosphorylates host TBC1D4, a GAP (GTPase-activating protein) for RAB14 (Ge et al., 2022). Phosphorylation of threonine-642 in TBC1D4 by PknG prevents its ability to activate GTP hydrolyzation in RAB14 (Ge et al., 2022). Thus, by interacting with RAB14, PknG directly phosphorylates TBC1D4, interfering with autophagosome maturation processes in the host cells (Figure 2). This illustrates another example of identifying host targets of bacterial effector kinases and their underlying molecular mechanisms through investigating affected pathways.

## Host substrates identified by screening for interacting partners of effector kinases

Typically, the interaction between an enzyme and substrate is transient. Nevertheless, some kinases and their substrates can have stable interactions, with the close substrate proximity facilitating phosphorylation, for example Gɑq and OSK1 for *Yersinia* YpkA and *Xanthomonas* XopC2, respectively. With feasibility of large-scale proteomic screens for protein interacting partners such as yeast two-hybrid analyses, and advances in mass spectrometry to identify proteins co-purified with bacterial effectors by pulldown assays (Figures 1C,D), host substrates that form stable complexes with effector kinases were identified.

### *Legionella* LegK2 interacts with and phosphorylates host ARP2/3 complex for ER recruitment

As an intracellular pathogen, *L. pneumophila* enters the host cells through phagocytosis and forms a *Legionella*-containing vacuole (LCV) where the bacterium replicates within host cells. The *L. pneumophila* T4SS effectors are essential for LCV formation by manipulating many host processes, such as phagocytosis, cytoskeleton rearrangement, and vesicle trafficking (Mondino et al., 2020). In addition to LegK1, LegK2 is one of the four *L. pneumophila* T4SS effectors that share primary sequence homology to eukaryotic serine/threonine kinases (Hervet et al., 2011). Notably, it has been shown that LegK2 and its kinase activity are required for optimal intracellular replication of *L. pneumophila* within amoeba hosts and recruitment of host ER to the LCV (Hervet et al., 2011).

To identify host factors interacting with LegK2, a yeast two-hybrid screen was utilized and subsequently revealed that host ARPC1B and ARP3 interact with LegK2 (Michard et al., 2015). An *in vitro* kinase assay showed that recombinant LegK2 phosphorylates ARPC1B/ARP3 proteins and immunoblotting demonstrated phosphorylated ARPC1B and ARP3 when co-expressed with LegK2 in HEK cells (Michard et al., 2015). ARPC1B and ARP3 are components of the actin nucleator ARP2/3 complex, and phosphorylation of ARPC1B is required for activation of actin nucleation (LeClaire et al., 2008; Michard et al., 2015), suggesting that LegK2 may interfere with host actin polymerization by phosphorylating ARPC1B/ARP3 (Figure 2). In addition, ectopically expressed LegK2 in human HeLa cells disrupts formation of actin filaments, and LegK2 inhibits actin polymerization around the LCV in amoeba (Michard et al., 2015). Despite detection of ARPC1B and ARP3 phosphorylation by LegK2 using immunoblotting, mass spectrometry failed to detect phosphorylation of ARPC1B or ARP3 (Michard et al., 2015), indicating that phosphorylation level of ARPC1B and ARP3 by LegK2 is relatively low in the cells. Therefore, further analysis is necessary to dissect whether LegK2 disrupts host actin filaments through phosphorylation or interaction with the host ARPC1B/ARP3 and to determine whether the impact of LegK2 on host cytoskeleton is mediated entirely by modulating ARP2/3 complex or interactions with other host factors. Nevertheless, through yeast two-hybrid screen for LegK2 interacting partners, the ARP2/3 components are identified as host targets of the kinase effector, which may contribute to manipulation of host cytoskeleton rearrangement.

### *Pseudomonas* HopBF1 phosphorylates host Hsp90 to prevent immune sensor activation

HopBF1 is a T3SS effector encoded by the plant pathogen *Pseudomonas syringae* and its homologs can be found in other plant and animal pathogens, such as *Ewingella americana* and *Burkholderia* spp. (Lopez et al., 2019). Despite limited primary sequence homology to eukaryotic kinases, HopBF1 exhibits predicted folding similar to protein kinases and aminoglycoside phosphotransferase. Crystal structure analyses with an *E. americana* HopBF1 homolog revealed that HopBF1 exhibits a minimal and atypical protein kinase folds (Lopez et al., 2019). Ectopic production of *P. syringae* HopBF1, but not its catalytic inactive variant, induces necrosis and collapse of leaf tissue in tobacco plants and inhibits cell growth in a yeast strain (Lopez et al., 2019), suggesting that HopBF1 targets a conserved host pathway in eukaryotic cells.

To identify host substrates of HopBF1, purified HopBF1 was incubated with yeast cell lysate and a phosphorylated protein product between 80-90kD was characterized (Lopez et al., 2019). Pulldown assays followed by mass spectrometry using yeast cell lysate containing inactive HopBF1 effector kinase revealed that yeast HSP82/HSC82, orthologs of human HSP90, co-precipitated with HopBF1 (Lopez et al., 2019), suggesting that HopBF1 interacts with eukaryotic HSP90. Consistent with the lysate-based phosphorylation experiment, *in vitro* kinase assays using purified proteins confirmed that HopBF1 directly phosphorylates serine-99 on yeast HSP90 (Lopez et al., 2019). Eukaryotic HSP90 functions as a molecular chaperone and plays an important role in plant immune responses against pathogens. Phosphorylation of host HSP90 by HopBF1 inhibits HSP90 ATPase and chaperone activity, leading to inactivation of plant nucleotide binding domain, leucine rich repeat containing proteins (NB-LRRs) that are important immune sensors against pathogens and interactors with plant HSP90 (Schulze-Lefert, 2004; Jones et al., 2016; Lopez et al., 2019; Figure 2). Because catalytically inactive HopBF1 also interacts with yeast HSP90 and many protein kinases are known clients of HSP90, it is likely that the interaction with host HSP90 stabilizes HopBF1 and HopBF1 mimics a host HSP90 client to interfere with the normal function of HSP90. Here, a technique of using a catalytic inactive effector kinase to co-precipitate host substrates, potentially through retaining the interactions with its substrates, lead to the identification of conserved HSP90 as a substrate of HopBF1. Together, this example illustrates the use of inactive enzymes to lock the transient enzyme-substrate interactions to facilitate substrate identification.

### *Yersinia* YpkA phosphorylates actin-interacting proteins to cripple host phagocytosis

Early studies reported that host actin interacts with YpkA, strongly activating the kinase activity of YpkA, indicating that actin is an allosteric activator for YpkA (Juris et al., 2000). Crystal structure analysis of a YpkA/actin complex revealed that YpkA binds to actin monomers through the kinase domain and C-terminal GDI binding domain (Lee et al., 2015, 2017). YpkA prevents actin polymerization by binding at actin subdomain 4 to sequester actin monomers (Lee et al., 2015). However, the region between actin subdomains 1 and 3 that normally mediates actin regulator protein binding remains unoccupied in the YpkA/actin complex, which may allow actin regulatory proteins to be incorporated into the complex.

To identify additional host proteins in the YpkA/actin complex, SILAC (Stable Isotope Labeling of Amino acids in Cell culture) was utilized to label mouse RAW264.7 cell lysate (Lee et al., 2015). The C^13^ heavy isotope labeled cell lysate was incubated with YpkA. After affinity purification of His-tagged YpkA from the labeled lysate, mass spectrometry was used to identify host proteins interacting with YpkA by measuring the enrichment ratio of proteins co-purified with His-tagged YpkA from heavy isotope labeled lysate compared to protein purified from bead-only, non-labeled lysate. This approach detected known interacting partners, such as actin and the Rho GTPase Rac2, and several additional actin-binding proteins, including profilin, EVL, VASP, mDia1, INF2, WASP, WIP, gelsolin, and cofilin1 (Lee et al., 2015), suggesting that YpkA, actin, and these actin-binding proteins form a ternary complex. It is possible that YpkA may use host actin as a “bait” to recruit these proteins. To test this hypothesis, *in vitro* kinase assays were used with purified YpkA, actin, and identified actin-binding proteins. The *in vitro* assays showed that YpkA phosphorylated VASP, EVL, mDia1, WASP, and gelsolin in an actin-dependent manner (Lee et al., 2015). Adding gelsolin domain 1 (G1) as a competitor for the binding region between actin subdomain 1 and 3 decreased phosphorylation of the actin-binding proteins (Lee et al., 2015), which supports that YpkA uses actin as an activator for its kinase activity as well as a bait to recruit host actin-associated proteins into the YpkA/actin kinase complex for further phosphorylation. *In vitro* polymerization assays revealed that phosphorylation of VASP by YpkA disrupts formation of actin filaments (Lee et al., 2015). In addition to phosphorylating host Gɑq, these findings suggest an alternative mechanism by which YpkA interferes with host cytoskeleton rearrangement (Figure 2). This study demonstrates an approach of combining pulldown assays and SILAC to identify unknown substrates of the kinase effector from host cell lysate.

## Phosphorylation proteomic screens for identifying host substrates of effector kinases

Substrate identification by testing components of host pathways affected by bacterial effector kinases may provide direct links between substrates and affected pathways, but may, however, be limited by the knowledge of components participating in the pathways and complicated by indirect effects from other pathways. Yeast two-hybrid screens and pulldown assays mainly identify host proteins that stably interact with the effector kinases but are likely to miss host substrates that have a transient interaction with the effector kinases. To achieve more comprehensive, non-biased screening, ATP analog-sensitive chemical genetic engineering and high-density protein microarrays combined with radioisotope ATP or non-radioactive ATPγS labeling have been developed and applied to identification of host substrates for bacterial effector kinases (Figure 1).

### Chemical genetic engineering: Identifying Otubain 1 as a substrate of *Yersinia* YpkA and HSP70 as a substrate of *Legionella* LegK4

Identification of substrates with the use of purified effector kinases and radioactive ATP in cell lysate is challenging because of the high level of background phosphorylation by host kinases. To overcome this challenge, a chemical genetic technique was successfully developed by modifying the gatekeeper residues in the ATP-binding pocket of kinases of interest, which allows the modified kinases to accept synthetic ATP analogs with bulky groups attached to the N^6^ position of the nucleotide (Shah et al., 1997). Because the bulky ATP analogs can only be used by the engineered analog sensitive (AS) kinase, but not wildtype kinases or endogenous kinases, only substrates of the AS kinase will be phosphorylated in the cell lysate upon co-incubation of the AS kinase and the bulky ATP analog (Figure 1E).

Based on this method, researchers mutated the methionine-211 residue in the kinase subdomain V of YpkA to glycine, which confers the YpkA^M211G^ capable of using bulky ATP analogs (Juris et al., 2006). Co-incubation of the AS YpkA, radiolabeled N^6^ ATP, and mouse J774 cell lysate revealed a ~ 36kD band was specifically phosphorylated by the AS YpkA (Juris et al., 2006). Gel-filtration and SDS-PAGE were used to isolate and separate the cytosolic fraction containing the 36 kD protein that was identified as otubain 1 (OTUB1), a deubiquitinating enzyme, by mass spectrometry (Juris et al., 2006). This phosphorylation event was further confirmed using *in vitro* kinase assays. Phosphorylation of OTUB1 by YpkA is actin-dependent, which is consistent to the model that YpkA uses actin as an allosteric activator (Figure 2). Interestingly, like other YpkA substrates, pulldown assays showed that OTUB1 also interacts with YpkA (Juris et al., 2006; Edelmann et al., 2010). It is likely that this phosphorylation modulates bacterial entry and uptake within the cell due to actin dependence and YpkA/OTUB1 complexing with the small GTPase RhoA (Edelmann et al., 2010).

Using radioactive bulky ATP analogs provides specificity and sensitivity to detect protein phosphorylation by AS kinases. However, it is still difficult to isolate and identify phosphorylated substrates from the cell lysate since the substrate is labeled by phosphates with radioisotopes that do not have significant chemical differences for affinity purification. To facilitate isolation of labeled substrates, a method that uses bulky ATPγS as phosphate donors was developed (Allen et al., 2007). The γ-thiophosphate of the bulky ATP analogs can be transferred to protein substrates by majority of kinases, and the sulfur atom on the γ-thiophosphate can serve as a molecular handle for chemical modifications, such as alkylation by *p*-nitrobenzylmesylate (PNBM). Furthermore, generation of an antibody that specifically recognizes the thiophosphate ester moiety conjugated on thiophosphorylated serine/threonine/tyrosine residues after PNBM alkylation allows for detection and purification of thiophosphorylated substrates by immunoblotting and immunoprecipitation, respectively (Allen et al., 2007).

Using the bulky ATPγS labeling technique, a host substrate of *L. pneumophila* effector kinase, LegK4, was recently identified. LegK4 contains primary sequence homology to eukaryotic serine/threonine kinases (Hervet et al., 2011) and is one of the *L. pneumophila* effectors that inhibit host protein translation when ectopically produced in HEK cells (Barry et al., 2013; Moss et al., 2019). Surprisingly, LegK4 can use N^6^ benzyl ATPγS as a phosphate donor without the need to engineer the gatekeeper residues in its ATP binding pocket. Co-incubation of purified LegK4, N^6^-benzyl ATPγS, and HEK cell lysate revealed two thiophosphorylated protein bands with molecular weights between 70-80kD (Moss et al., 2019). After immunoprecipitation of the thiophosphorylated proteins and mass spectrometry, it was discovered that LegK4 directly phosphorylates the host HSP70 family proteins, HSC70, HSP72, and Bip, on the threonine-495 of HSC70/HSP70 (threonine-518 of Bip; Moss et al., 2019; Figure 2). HSP70 family proteins contain ATPase activity and function as chaperones that regulate protein folding and homeostasis. The phosphorylation of HSC70 by LegK4 results in reduced ATPase activity and protein folding activity *in vitro*, and ectopic expression of LegK4 in mammalian cells causes suppression of the unfolded protein response and protein translation (Moss et al., 2019), phenomena that likely result from phosphorylation of HSC70 by LegK4.

### *Legionella* LegK7 phosphorylates MOB1, the scaffold protein in the host hippo pathway

*L. pneumophila* LegK7 is a T4SS effector kinase that is conserved in many sequenced *Legionella* genomes. Unlike other previously known *L. pneumophila* effector kinases, LegK1 to LegK4, LegK7 has limited primary sequence homology to eukaryotic serine/threonine kinases but a high degree of protein folding similarity to eukaryotic kinases based on HHPred prediction (Lee and Machner, 2018). To identify host substrates of *L. pneumophila* effector kinases, a high throughput screen platform that combines the ATPγS labeling technique and a high-density protein microarray was developed (Lee and Machner, 2018). Purified LegK7 proteins were co-incubated with ATPγS on a protein microarray containing ~10,000 purified human proteins, and thiophosphorylated protein spots were then alkylated by *p*-nitrobenzylmesylate (PNBM; Lee and Machner, 2018; Figure 1F). This modification can be detected by the specific antibody that recognizes the thiophosphate ester moiety as mentioned in the earlier section followed by a fluorescence conjugated secondary antibody. This platform provides fast screening of thousands of human proteins for potential direct substrates *in vitro* with advantages of high sensitivity based on antibody-fluorescence detection and use of non-radioactive ATP analogs without engineering the kinase. Screens using this platform revealed that LegK7 directly phosphorylates host MOB1 (Mps one binder kinase activator-like 1), a key scaffold protein of the conserved Hippo pathway, to alter host gene expression to promote *L. pneumophila* infection (Lee and Machner, 2018; Figure 2).

The eukaryotic Hippo pathway plays important roles in tissue development and cancers by regulating cell growth and proliferation (Harvey et al., 2013). The scaffold protein, MOB1, is phosphorylated by the Hippo kinase MST1 in humans, on threonine-12 and-35, which allows phosphorylated MOB1 to interact with the LATS/NDR kinases and activate their kinase activity (Praskova et al., 2008). Like host MST1, *L. pneumophila* LegK7 phosphorylates threonine-12 and-35 of MOB1 (Lee and Machner, 2018). Unexpectedly, kinase activity of LegK7 was elevated upon co-incubation with MOB1 in an *in vitro* kinase assay, suggesting that MOB1 is not only a substrate, but also an activator of LegK7 (Lee et al., 2020). Structural analyses of a LegK7/MOB1 complex confirmed that MOB1 interacts with the N-terminal non-kinase domain and the kinase domain of LegK7 at an interface opposite the catalytic cleft and that disruption of the interaction abolished upregulation of LegK7 kinase activity (Lee et al., 2020). Furthermore, the LegK7/MOB1 complex utilizes the intrinsic activity of MOB1 N-terminal extension to recruit downstream substrates, such as the host transcription factor YAP1, into the kinase complex for phosphorylation (Lee et al., 2020). Thus, *L. pneumophila* LegK7 is a functional chimera of two core kinases, MST1 and LATS/NDR, of the host hippo pathway. Like *Yersinia* YkpA exploits host actin, LegK7 uses its MOB1 substrate as an allosteric activator and recruiter for downstream host substrates.

### *Escherichia coli* NleH1/NleH2 phosphorylate host EPS8 to modulate formation of intestinal microvilli

Enteropathogenic *E. coli* encode two T3SS effectors, NleH1 and NleH2, that are on different genomic loci, but highly similar with 84% amino acid sequence identity (Gao et al., 2009). NleH1/NleH2 contain kinase domains that are similar to T3SS effector kinases in other enteropathogenic bacteria, including *Shigella flexneri* OspG (Tobe et al., 2006), *Citrobacter rodentium* NleH (García-Angulo et al., 2008), and *Vibrio parahaemolyticus* VopG (Plaza et al., 2021). NleH1/NleH2 can prevent host apoptosis *via* their ability to interact with Bax inhibitor-1 (BI-1), an anti-apoptotic protein in host cells. However, the anti-apoptotic effect does not require the kinase activity of the NleH effectors (Hemrajani et al., 2010), suggesting that BI-1 or other host proteins controlling apoptosis are not phosphorylated by the NleH effectors. Studies have shown that another host pathway affected by the NleH effectors is the NFκB signaling pathway (Gao et al., 2009; Wan et al., 2011; Pham et al., 2013). NleH1 suppresses phosphorylation of ribosomal protein S3 (RPS3), a component of the NFκB complex, by altering substrate specificity of IκB kinase β (IKKβ; Wan et al., 2011). Moreover, NleH1 interacts with RPS3 (Gao et al., 2009). Interestingly, NleH1 kinase activity is essential for altering substrate specificity of IKKβ and suppressing RPS3 phosphorylation (Wan et al., 2011); however, both IKKβ and RPS3 are not substrates of NleH1.

A screen for host substrates of NleH1 was performed by co-incubation of purified NleH1 with radioactive γ-^33^P ATP on a high-density human protein microarray (Pham et al., 2013). This technique is the same in principle as the LegK7 substrate screen discussed in the previous section, and the main difference is the detection methods for phosphorylated host proteins on the microarrays. The NleH1 screen identified v-Crk sarcoma virus CT10 oncogene-like protein (CRKL), EGF receptor kinase pathway substrate 8-like protein 2 (EPS8L2), and microtubule-associated protein RP/EB family member 1 (MAPRE1) are NleH1 candidate substrates (Pham et al., 2013). Separate *in vitro* kinase assays confirmed that NleH1 directly phosphorylates CRKL, and pulldown assays revealed that these two proteins stably interact (Pham et al., 2013). While a catalytic inactive NleH1 effector kinase failed to interact with CRKL, it is unclear whether this interaction is mediated by CRKL phosphorylation or autophosphorylation of NleH1. Nevertheless, knocking down CRKL expression by siRNA reduces impacts of NleH1 on RPS3, which is possibly due to that CRKL can interact with IKKβ (Pham et al., 2013).

Recently, another attempt to identify host substrates for NleH1 and NleH2 was conducted using label-free phosphoproteome analyses (Pollock et al., 2022) in infected host cells (Figure 1G). Human colon cancer cells, HT-29, were infected with wildtype enteropathogenic *E. coli* or mutants with the NleH effector genes deleted. Phosphorylated peptides were isolated by TiO2-beads from the infected cell lysate, and comparative mass spectrometry was used to quantitate phospho-peptides that were enriched in the host cells infected with wildtype bacteria. This assay revealed increased phosphorylation in multiple host proteins. Surprisingly, EGF receptor kinase pathway substrate 8 (EPS8) family proteins, including EPS8, EPS8L1 and EPS8L2, had the highest levels of phosphorylation (Pollock et al., 2022). EPS8L2 was previously identified as a putative substrate of NleH1 in the screen using human protein microarrays (Pham et al., 2013), suggesting that the EPS8 family proteins are likely genuine substrates of NleH1. Serine-775 of EPS8 was confirmed as a specific phosphorylation site by NleH1 and NleH2, and phosphorylation of this residue affected EPS8 bundling activity and cellular localization in host cells (Pollock et al., 2022). Moreover, the NleH effectors interact with EPS8, and this interaction, together with the kinase activity, are required for suppressing EPS8 bundling (Pollock et al., 2022; Figure 2).

## Effector kinases with unknown host targets

Thus far, a plethora of techniques to identify host substrates of bacterial effector kinases, such as host pathway characterization, protein–protein interactions, and ATP analog-based proteomic screen techniques, have proven to be applicable. Bacteria are constantly evolving to target new host pathways and avoid host defenses. These bacterial effector kinases possess intriguing abilities to phosphorylate versatile host substrates and utilize host factors as allosteric activators. However, while there exists other bacterial effector kinases with no known host substrates, recent advances may allow for the identification of previously challenging kinase substrates and their effects in host cell signaling.

### Shigella OspG and host NFκB pathway

*Shigella flexneri* T3SS effector OspG shares primary sequence homology with eukaryotic kinases and *E. coli* NleH1/NleH2 (Zhou et al., 2013), as discussed in the previous section. Using a yeast two-hybrid screen, host E2 ubiquitin conjugating enzymes, such as UbcH5c and UbcH5b, were revealed to interact with OspG, and *in vitro* co-purification assays showed that OspG specifically interacts with ubiquitin-conjugated UbcH5b (Kim et al., 2005). Ubiquitin-conjugated UbcH5c stimulated autophosphorylation of OspG, and structural analysis of OspG/UbcH5c ~ ubiquitin complex revealed that OspG contacts both UbcH5c and ubiquitin (Pruneda et al., 2014). Consequently, binding with UbcH5c ~ ubiquitin stabilizes OspG in an active conformation, showing that UbcH5c ~ ubiquitin complex is an allosteric activator for OspG (Pruneda et al., 2014). However, no UbcH5c phosphorylation was observed in an *in vitro* kinase assay, indicating that UbcH5c is not a substrate of OspG.

UbcH5c is one of the components of the SCF complex, which mediates ubiquitination of phosphorylated IκB for subsequent proteasomal degradation. Ectopic expression of wild type OspG, but not catalytically inactive OspG, prevents degradation of IκBα and suppresses expression of an NFκB-driven luciferase reporter in TNFα-treated HEK cells (Kim et al., 2005), suggesting that OspG inhibits the host NFκB pathway. Consistently, human HeLa cells infected with an *S. flexneri ospG* mutant had faster IκBɑ degradation than cells infected with wild type *S. flexneri*, and rabbits infected with *ospG* knockout *S. flexneri* displayed more severe inflammation in the intestinal ligated ileal loop model (Kim et al., 2005). While it is clear that OspG affects the NFκB pathway and modulates host inflammatory responses during infection, host substrates of OspG remain unidentified.

### Legionella Lem28 effector kinase is activated by host inositol Hexakisphosphate

*L. pneumophila* effector, Lem28, is encoded by the gene *lpg2603*, and its homologs can be found in other *Legionella* species. Functional motif searches suggest that Lem28 contains a phosphatidylinositol-4-phosphate (PI4P) binding domain in the C-terminus and a putative tyrosine kinase domain in the N-terminus (Burstein et al., 2016). Like other *L. pneumophila* PI4P-binding domain containing effectors, SidM and Lpg1101, the PI4P binding activity is required for localizing Lem28 to the host plasma membrane. In infected host cells, Lem28 is secreted by the T4SS and associated with the *Legionella*-containing vacuole (LCV); however, Lem28 without PI4P-binding activity still localizes on the LCV. When ectopically produced in yeast, Lem28 suppresses yeast growth and requires the PI4P binding domain (Hubber et al., 2014), suggesting that this effector disrupts a conserved eukaryotic pathway. Testing recombinant Lem28 kinase activity using *in vitro* assays did not detect autophosphorylation of the effector kinase or phosphorylation of a pseudo-substrate, myelin basic protein (MBP). Because many bacterial effectors use host factors to activate their enzymatic activities, the addition of host factors into the kinase assays lead to the interesting discovery that Lem28 co-incubated with inositol hexakisphosphate (IP6) phosphorylates MBP (Sreelatha et al., 2020). Biochemical and structural analyses also confirmed that IP6 interacts with Lem28 and that this interaction stabilizes Lem28 and facilitates ATP-binding. These assays demonstrate that IP6 is an allosteric activator for Lem28 kinase activity, which is an additional example of a bacterial effector being activated by a trans-kingdom activator from the host cells (Anderson et al., 2015). Although substrate identification *via* isolating host proteins interacting with the effector was attempted (Anderson et al., 2015), host proteins phosphorylated by Lem28 are still unknown. Thus, substrate screening from host cell lysate using phosphoproteomic mass spectrometry or a high-throughput microarray with Lem28 co-incubated with IP6 are potential options to address the transient interactions between this effector kinase and its host substrates.

### Vibrio VopG contains key catalytic residues similar to NleH effector kinases

*Vibrio parahaemolyticus* uses a T3SS to secrete nine effector proteins into host cells during infection to target actin cytoskeleton and inflammatory responses, although more effectors are continuously being identified (Plaza et al., 2021). The newly identified *V. parahaemolyticus* VopG is a homolog of *Vibrio cholerae* VopG, and exists in many *Vibrio* species (Plaza et al., 2021). Using HHPred and pGenTHREADER to analyze *V. parahaemolyticus* VopG, sequence and structural similarity to the NleH family of T3SS effector kinases in *E. coli* and *S. flexneri* was found (Plaza et al., 2021), suggesting a potential role for VopG in phosphorylation of host substrates. NleH proteins display functional similarity in their serine/threonine kinase domain to eukaryotic kinases and promote intracellular replication by targeting NFκB signaling (Hodgson and Wan, 2016), preventing programmed cell death and defensive immune responses. Recently, VopG’s sequence was analyzed and discovered to contain key catalytic residues and motifs required for kinase activity and shares specific residues with the NleH family of proteins involved in phosphorylation (Plaza et al., 2021). However, dissimilar to NleH proteins, VopG did not appear to suppress apoptosis or IL-8 production, suggesting a novel, but unidentified role for VopG in host cells (Plaza et al., 2021). While VopG likely acts as an effector kinase based its sequence and structural similarity, its activity and host substrate are both uncharacterized, lending to the importance of multiple approaches for substrate identification and pathway interactions.

## New approaches for effector kinases and host substrate identification

**Figure 3:**
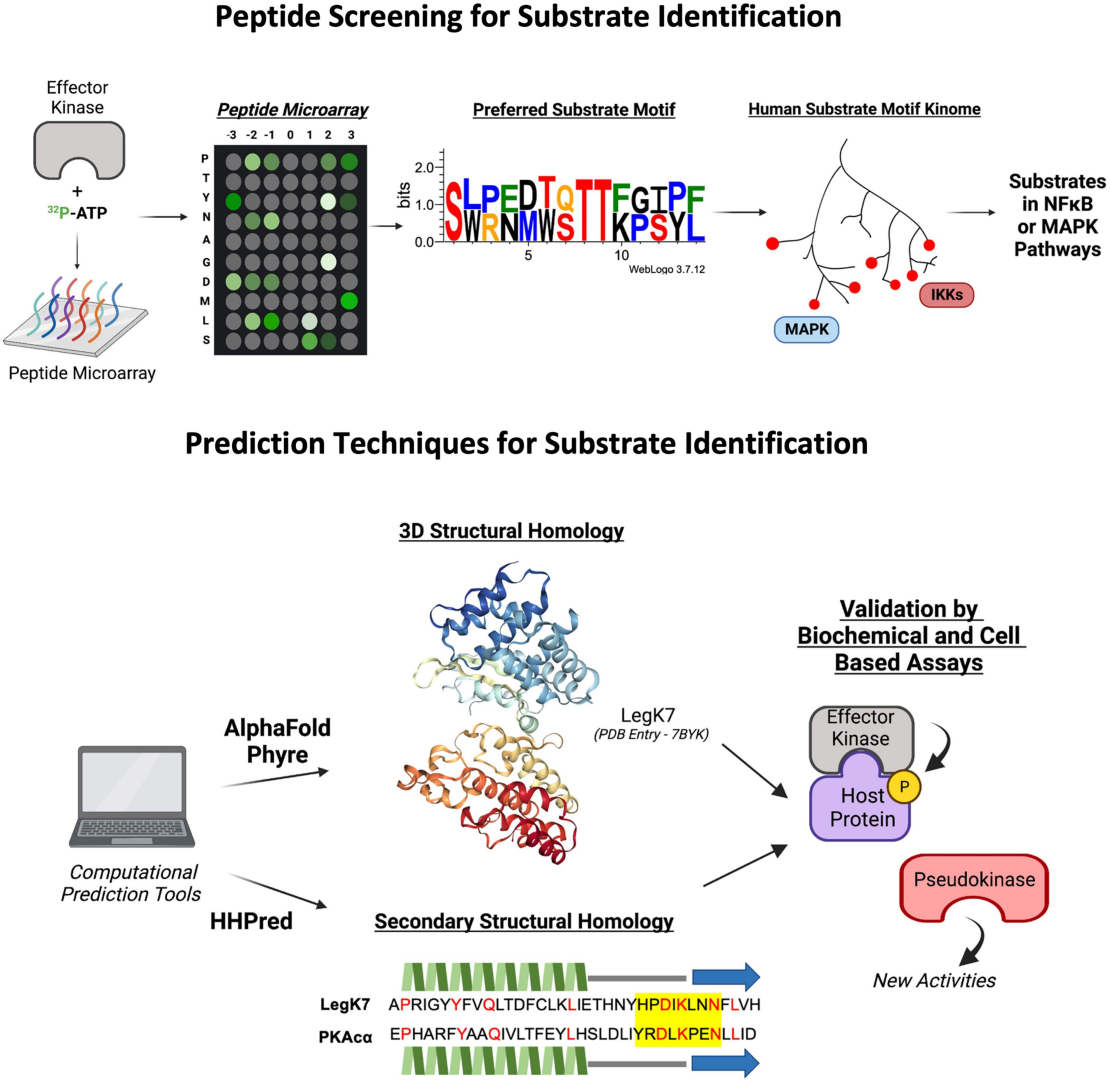
Novel predictive tools and applications, such as peptide screens and computational structure and folding algorithms in identifying putative effector kinases and their host substrates. LegK7 crystal structure from Protein Database (PDB Entry Number: 7BYK). Protein motif sequence generated through WebLogo3. The predicted secondary structure alignment of PKAcɑ and LegK7 is shown. Beta strands are represented by blue arrows, helices are represented by green jagged lines, loops are represented as straight gray lines, catalytic motif is highlight yellow, and identical residues are indicated by red color.

Thus far, we have described a multitude of techniques that have been used to identify bacterial effector kinases and their host substrates. There are, however, other approaches that may also be applicable identification methods for effector kinases and host substrates. For example, similar to the ATPγS protein microarray used to screen the substrates of LegK7 as previously described, peptide microarrays may also be utilized for identification of peptide sequences or motifs that are preferred by bacterial effector kinases. This method relies on the phosphorylation screening of a microarray chip containing short amino acid motifs co-incubated with the kinase of interest (Xue and Tao, 2013; Figure 3). This approach has been used to identify host substrates of a kinase from Kaposi’s sarcoma-associated herpesvirus (KSHV), vPK (Bhatt et al., 2016). The screening revealed that vPK preferentially phosphorylates peptide motifs overlapping with the motifs phosphorylated by the host kinase S6KB1, suggesting that vPK might also target the same substrates. Further analysis demonstrated that vPK functionally mimics S6KB1 to phosphorylate ribosomal S6 and promote protein translation in host cells (Bhatt et al., 2016). While this method is relatively less expensive than full protein microarrays, it mainly provides information related to the preferred substrate sequence motifs of a kinase and does not identify a direct substrate interaction. Any proteins that contain the sequence motifs could be potential substrates and will require additional validation. However, the recent construction of a kinome database of mammalian kinase substrate sequences may provide further aid in narrowing the search to specific substrate families or pathways that may be targeted by the effector kinase through shared preference in substrate motifs (Johnson et al., 2023; Figure 3).

With the advancements in artificial intelligence (AI)- and neural network-based algorithms to compute complex biological interactions, predictive computational tools may prove beneficial in the identification of effector kinases based on structural and folding homology to known kinases (Figure 3). For example, AlphaFold, HHPred, and Phyre2 are complex *in silico* applications capable of predicting three-dimensional protein structure and folding probabilities based on the protein’s amino acid sequence, and in the case of AlphaFold, to near atomic accuracy even without any homologous structure (Hildebrand et al., 2009; Kelley et al., 2015; Jumper et al., 2021). Applications such as AlphaFold have recently been used to characterize the conformational properties of eukaryotic kinases such as diacylglycerol kinases (DGKs; Aulakh et al., 2022) and may aid in the identification of bacterial effector kinases through structural and folding pattern predictions. *In silico* techniques are constantly evolving, such as the AlphaFold partner algorithm, AlphaFill, which uses sequence and structural information to fill in missing small molecules such as ATP (Hekkelman et al., 2022). These new technologies may further advance the ability to predict putative effector kinases for following experimental validation and functional studies.

## Conclusion

Kinases play a central role in signal transduction in living cells. Many bacterial pathogens deliver effector kinases into their host cells to interfere with signaling and subvert immune responses. Studying bacterial effector kinases has revealed how host responses, such as phagocytosis and inflammatory gene expression are manipulated. Moreover, investigation of bacterial kinases has led to the discovery of new cellular components and pathways, including heat shock chaperone proteins and the Hippo pathway, which serve as targets for bacterial effector kinases and play new roles in microbial pathogenesis. With the increasing number of sequenced bacterial genomes, more effector kinases are expected to be discovered. For example, a genomic analysis that included 58 *Legionella* genomes showed that these *Legionella* species encode more than 18,000 T4SS effectors and eukaryotic protein kinase motifs are the second most abundant functional motifs found in these effectors based on primary sequence homology (Gomez-Valero et al., 2019). In addition, like *Pseudomonas* HopBF1 and *Legionella* LegK7, effector proteins that have limited sequence homology but high folding similarity to eukaryotic kinases have been discovered with advances in structural prediction tools, which opens a new class of effector kinases that remain to be explored. More interestingly, a *Legionella* effector SidJ, which also shares structural similarity and borderline sequence homology to eukaryotic kinases, has recently been shown to be a “pseudokinase” with novel enzyme activity capable of polyglutamylating the *Legionella* SidE family of effectors (Black et al., 2019). The discovery of pseudokinases presents another layer of complexity in identifying effector kinases and their substrates, due to these effectors being characterized as catalytically inactive in protein phosphorylation, but with structural or sequence homology to known kinases (Poh et al., 2008). For example, *Pseudomonas syringae* SelO shares structural and folding homology with eukaryotic kinases, but has been shown to catalyze AMPylation (Sreelatha et al., 2018; Pon et al., 2023). SidJ and SelO, among other microbial proteins, demonstrate that sequence or structural homology is not sufficient to categorize effectors as kinases, and their biochemical activity must be experimentally tested. Previously, SidJ was shown to modify another *L. pneumophila* effector, SdeA, and mass spectrometry analysis revealed glutamylation of sdeA as a new activity of SidJ (Black et al., 2019). *In vitro* assays using ɑ-P32 labeled ATP instead of γ-P^32^ ATP for protein phosphorylation uncovered self-AMPylation of SelO, and further mass spectrometry analysis detected its AMPylation activity (Sreelatha et al., 2018). Some effectors with putative kinase domains, such as *V. parahaemolyticus* VopG, have not been experimentally validated for their biochemical activity. Thus, it is important to examine whether VopG may possess another activity other than protein phosphorylation. While new biochemical activities of effectors that share structural and sequential homology to kinases are exciting, challenges arise in predicting and determining their new activities. These discoveries highlight the diverse and novel functions of the effector kinase superfamily in bacterial pathogenesis.

Throughout this review, we have described multiple techniques to identify many novel bacterial effector kinase substrates (Figure 1). Each of these methods presents inherent advantages and limitations that require additional validation, and different approaches may lead to the discovery of different sets of kinase substrates, highlighting the versatile impact of effector kinases on host signaling (Figure 2). It is evident that many biological processes in host cells are highly regulated by protein phosphorylation. Although pathogens secrete phylogenetically distinct effector kinases and target different host substrate proteins, many of them converge on the same pathway with similar outcomes in the host cell. For example, SteC from *S. enterica* and LegK2 from *L. pneumophila* phosphorylate different host proteins but both result in cytoskeletal rearrangement. Conversely, effectors from different organisms that share a high degree of sequence similarity, such as NleH1/2 and OspG, target different host pathways and result in different cellular outcomes. These examples highlight the importance of protein phosphorylation in the host and the diversity in substrate targeting by effector kinases during infection. Ultimately, advancements in the discovery and characterization of effector kinases and their function in subverting host defenses to promote bacterial survival and replication allows for novel antibiotic targets and therapeutic treatments.

## Author contributions

BL, SQ, and P-CL wrote and edited the manuscript. All authors reviewed and approved the final version of the manuscript.

## Funding

This work and research in the Lee lab were supported by Wayne State University Startup Funds and the University Research Grant 2020–21 from Wayne State University.

## Conflict of interest

The authors declare that the research was conducted in the absence of any commercial or financial relationships that could be construed as a potential conflict of interest.

## Publisher’s note

All claims expressed in this article are solely those of the authors and do not necessarily represent those of their affiliated organizations, or those of the publisher, the editors and the reviewers. Any product that may be evaluated in this article, or claim that may be made by its manufacturer, is not guaranteed or endorsed by the publisher.
